# Reflecting on Mirror Mechanisms: Motor Resonance Effects during Action Observation Only Present with Low-Intensity Transcranial Magnetic Stimulation

**DOI:** 10.1371/journal.pone.0064911

**Published:** 2013-05-28

**Authors:** Michela Loporto, Paul S. Holmes, David J. Wright, Craig J. McAllister

**Affiliations:** 1 Institute for Performance Research, Manchester Metropolitan University, Manchester, United Kingdom; 2 Department of Life Sciences, University of Roehampton, London, United Kingdom; 3 Aston Brain Centre, School of Life and Health Sciences, Aston University, Birmingham, United Kingdom; Cardiff University, United Kingdom

## Abstract

Transcranial magnetic stimulation (TMS) studies indicate that the observation of other people's actions influences the excitability of the observer's motor system. Motor evoked potential (MEP) amplitudes typically increase in muscles which would be active during the execution of the observed action. This ‘motor resonance’ effect is thought to result from activity in mirror neuron regions, which enhance the excitability of the primary motor cortex (M1) via cortico-cortical pathways. The importance of TMS intensity has not yet been recognised in this area of research. Low-intensity TMS predominately activates corticospinal neurons indirectly, whereas high-intensity TMS can directly activate corticospinal axons. This indicates that motor resonance effects should be more prominent when using low-intensity TMS. A related issue is that TMS is typically applied over a single optimal scalp position (OSP) to simultaneously elicit MEPs from several muscles. Whether this confounds results, due to differences in the manner that TMS activates spatially separate cortical representations, has not yet been explored. In the current study, MEP amplitudes, resulting from single-pulse TMS applied over M1, were recorded from the first dorsal interosseous (FDI) and abductor digiti minimi (ADM) muscles during the observation of simple finger abductions. We tested if the TMS intensity (110% vs. 130% resting motor threshold) or stimulating position (FDI-OSP vs. ADM-OSP) influenced the magnitude of the motor resonance effects. Results showed that the MEP facilitation recorded in the FDI muscle during the observation of index-finger abductions was only detected using low-intensity TMS. In contrast, changes in the OSP had a negligible effect on the presence of motor resonance effects in either the FDI or ADM muscles. These findings support the hypothesis that MN activity enhances M1 excitability via cortico-cortical pathways and highlight a methodological framework by which the neural underpinnings of action observation can be further explored.

## Introduction

Observing and understanding other people's actions is crucial to our communication and social interactions. By observing others, we create an internal representation of that perceived action and use this information to predict future behaviours [1]. Action observation has also been successfully incorporated into clinical stroke rehabilitation programmes, significantly improving motor function, more so than physical therapy alone (e.g., [2]–[4]). The neural mechanisms underlying these processes are therefore of great interest. An action observation network, also termed mirror neuron system (MNS), which includes the premotor cortex, parietal areas and the superior temporal sulcus [5] has been proposed as the system responsible for many aspects of social cognition [6]. This network is thought to allow visual information from observed actions to be mapped onto the observer's motor system, causing the observer's brain to simulate the observed action [7]. Neuroimaging studies have shown similar neural representations between observation and execution (for a review see [8], [9]), reinforcing the proposal of a human MNS.

Transcranial magnetic stimulation (TMS) applied over the primary motor cortex (M1) elicits motor evoked potentials (MEPs) in the contralateral hand muscles, the amplitude of which provide a measure of corticospinal excitability at the time of stimulation. This method has been widely used to investigate the effects of action observation on the human motor system. For example, Fadiga et al. [10] first demonstrated that the observation of hand and arm actions increased corticospinal excitability, but only in those muscles used to perform the observed action. This muscle-specific motor facilitation effect, hereafter referred to as motor resonance, has been replicated repeatedly (for reviews see [11], [12]), and is proposed to result from activation of mirror neurons in premotor cortex regions facilitating motor cortex excitability through cortico-cortical connections [11], [13].

The accepted mechanism, by which TMS activates M1 to elicit descending volleys, and subsequently MEPs, is termed the D- and I-wave hypothesis [14], [15]. Low-intensity TMS primarily elicits I-waves, which result from ‘indirect’ trans-synaptic activation of corticospinal neurons. In contrast, high-intensity TMS predominately elicits D-waves, which result from the ‘direct’ activation of corticospinal axons. Due to the different sites of stimulation, I-wave amplitudes are more responsive to factors that influence cortical excitability. Consequently, MEP amplitudes elicited by low-intensity TMS are considered more representative of M1 excitability at the time of stimulation than MEP amplitudes elicited by high-intensity TMS [15]. It therefore follows that if MN activity acts on M1 excitability through cortico-cortico projections [13], then motor resonance effects are most likely to be detected with the use of low-intensity TMS. Despite having important implications for the mechanisms responsible for generating MEPs, the choice of stimulation intensity has not yet received consideration in the action observation literature. Motor resonance effects have been reported using a wide range of stimulation intensities from low-intensities of 110% resting motor threshold (RMT; e.g., [16], [17]), up to high-intensities of 130% RMT (e.g., [18], [19]). However, as yet, no study has performed a direct comparison of the size of this effect following the use of both low and high stimulation intensities.

Related to the choice of TMS intensity, the choice of optimal scalp position (OSP), or ‘motor hotspot’, is another parameter in need of further investigation. To establish the presence of motor resonance effects, MEPs need to be recorded from multiple muscles in response to a single stimulation (e.g., EMG recordings from finger and wrist muscles during observation of a reach and grasp action). This is typically achieved by fixing the stimulating coil over the OSP, and eliciting large short-latency MEP amplitudes in one of the target muscles. As a result of this method, secondary muscles that have different spatial representations within the motor cortex are not being stimulated at their respective OSPs. This affects the muscle's threshold and the relative intensity of stimulation applied to each muscle's cortical representation will differ. Again, to the best of our knowledge, no study has yet tested whether the presence of motor resonance effects are influenced by the choice of OSP. It is, therefore, an open question as to whether the commonly reported failure to detect MEP facilitations in secondary muscles is an artefact of the stimulation method.

The current study explored if the observation of simple hand actions produced an increase in corticospinal excitability that was specific to those muscles which would be active when performing the observed action. For the reasons outlined above, we also tested whether the choice of stimulation intensity and OSP determined the magnitude of the motor resonance effect. Our main hypothesis was that the motor resonance effect would be more prominent with the use of the low intensity TMS as compared to high intensity TMS.

## Materials and Methods

### Participants

Seventeen healthy volunteers (four females), aged 18 to 24 years (mean age 19.6 years) participated in experiment 1, and nineteen healthy volunteers (six males), aged 18 to 45 years (mean age 24.1 yrs) participated in experiment 2. All participants gave their written informed consent and were naïve to the purpose of the experiment. The TMS Adult Safety Screen [20] was used to identify any participants who may have been predisposed to possible adverse effects of the stimulation. No participants were excluded from the study based on their questionnaire responses and no discomfort or adverse effects during TMS were reported. All participants were right-handed as assessed by the Edinburgh Handedness Inventory [21]. The protocol was approved by a Departmental Ethics Committee at Manchester Metropolitan University and conducted in accordance with the Declaration of Helsinki (2008).

### Equipment and Protocol

#### Electromyographic Recordings

Electromyographic (EMG) recordings were collected from the first dorsal interosseous (FDI) muscle of the right hand in experiment 1, and simultaneously from the FDI and abductor digiti minimi (ADM) muscles of the right hand in experiment 2, using bipolar, single differential, surface EMG electrodes (DE-2.1, Delsys Inc, Boston, MA). The electrodes comprised of two 10 mm×1 mm silver bar strips, spaced 10 mm apart, recorded with a bandwidth 20 Hz to 450 kHz, 92 dB common mode rejection ratio, and >10^15^ Ω input impedance. The electrodes were placed over the belly of the muscles and a reference electrode was placed over the ulnar process of the right wrist. The EMG signal was recorded using Spike 2 version 6 software (Cambridge Electronic Design (CED), Cambridge), received by a Micro 1401 analogue-digital converter (CED).

#### Transcranial Magnetic Stimulation

TMS was performed with a figure-of-eight coil (mean diameter of 70 mm) connected to a Magstim 200^2^ magnetic stimulator (Magstim Co., Whitland, Dyfed, UK) which delivered monophasic pulses with a maximum field strength of 2.2 Tesla. The coil was held in a fixed position, using a mechanical arm, over left M1. The coil was orientated so that the flow of induced current in the brain travelled in a posterior-anterior direction, perpendicular to the central sulcus; the optimal orientation for achieving indirect trans-synaptic activation [22] with a Magstim 200^2^ stimulator. The OSP was defined as the site which produced MEPs of the greatest amplitude with a stimulation intensity of 60% maximum stimulator output. The OSP was marked on a tightly fitting polyester cap on the participant's head to ensure a constant location throughout the experiment. The abbreviations FDI-OSP and ADM-OSP refer to when the TMS coil was at the optimal position for obtaining MEPs from the FDI and ADM muscles respectively. Finding the OSP at an intensity of 60% stimulator output is sensible, as it produces large short-latency MEPs in most people, and is common in TMS action observation research (e.g., [23], [24], [25]) The intensity was then reduced or increased as appropriate until resting motor threshold (RMT) was achieved. Resting motor threshold (RMT) was defined as the minimum stimulation intensity that elicited peak-to-peak MEP amplitudes greater than 50 µv in at least 5 out of 10 trials [26]. When the TMS coil was placed over the FDI-OSP, RMT was calculated using the MEP amplitude recorded from the FDI muscle;(hereafter referred to as FDI-RMT); when the coil was placed over the ADM-OSP, RMT was calculated using the MEP amplitude recorded from the ADM muscle (hereafter referred to as ADM-RMT).

### Experimental Procedures

Participants were seated in a dimly illuminated room in a comfortable chair with their elbows flexed at 90° and their hands placed in a relaxed position on a table in front of them. The participant's head was rested on a chin and head rest to restrict movement. A 37 inch Panasonic LCD television screen (resolution, 1024×768 pixels; refresh frequency, 60 Hz) was positioned at a distance of 40 inches from the participant. Participants were requested to refrain from any voluntary movement and to attend to the stimuli presented on the television screen. Blackout curtains ran along either side of the table and behind the screen to eliminate any distractive visual stimuli in the room.

**Figure 1:**
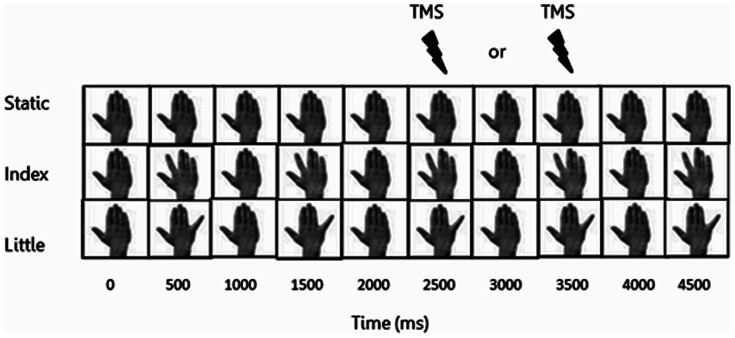
Three different videos used in this study. Experiment 1 consisted of: (i) static hand and; (ii) index-finger movements. Experiment 2 consisted of: (i) static hand; (ii) index-finger movements and; (iii) little finger movements. One TMS pulse was delivered per video at either 2500 or 3500 ms after video onset.

Participants observed the following three types of video during this study (see Figure 1). The first video, labelled STATIC, showed the dorsal view of a hand resting in a prone position. The second video, labelled INDEX, showed the same hand performing five abductions of the index-finger. The third video, labelled LITTLE, showed the same hand performing five abductions of the little-finger. All videos were of five second duration and were recorded using both male and female hands.

### Experimental protocols

Experiment 1 tested whether the TMS intensity affected the magnitude of the motor facilitation produced by action observation. The protocol of experiment 1 consisted of a single experimental session during which the TMS coil was positioned over the FDI-OSP. Participants observed 40 STATIC and 40 INDEX videos, which were presented in a random order and split across four blocks. A single pulse of TMS was applied at either 2500 ms or 3500 ms after the onset of each video. These timings corresponded to the point of maximal abduction in the INDEX video. MEP amplitudes were recorded from the FDI muscle. TMS was applied with a low intensity of 110% FDI-RMT during the first 10 trials of each block and with a high intensity of 130% FDI-RMT during the second 10 trials of each block. There was an inter-trial interval of six seconds and a two minute rest period between blocks.

Experiment 2 tested whether the choice of OSP affected the magnitude of the motor facilitation produced by action observation. The protocol was performed over two experimental sessions, separated by at least 24 hours. In each session, participants observed 36 STATIC, 36 INDEX and 36 LITTLE videos, which were presented in a random order across three blocks. There was an inter-trial interval of six seconds and a two minute rest period between blocks. A single pulse of TMS was applied at either 2500 ms or 3500 ms after the onset of each video. These timings corresponded to the point of maximal abduction in both the INDEX and LITTLE videos. MEP amplitudes were recorded from both the FDI and ADM muscles. The TMS coil was positioned over the FDI-OSP in one session and positioned over the ADM-OSP in the other (order randomised across participants). During the FDI-OSP session, the TMS intensity was set to 110% FDI-RMT. During the ADM-OSP session the TMS intensity was set to 110% ADM-RMT.

### Data Analysis

A pre-stimulus recording of 200 ms was used to check for the presence of EMG activity before the TMS pulse was delivered. Individual trials in which the peak-to-peak amplitude of the baseline EMG activity was 2.5 SD higher than the mean baseline EMG activity of each participant were discarded from further analysis since it may have influenced the amplitude of the subsequent MEP. As a result, 1.6% of low intensity trials and 1.9% of high intensity trials in experiment 1, and 2.2% of FDI-OSP trials and 2.1% of ADM-OSP trials in experiment 2, were discarded.

The peak-to-peak MEP amplitude was first measured from every individual trial and then the mean MEP amplitude was calculated for each observation condition (Tables 1 and 2). Due to the large inter-participant variability in absolute MEP amplitudes, these data were normalised using the *z*-score transformation (e.g., [10], [24]). In experiment 1, the normalised MEP amplitudes recorded from the FDI muscle were analysed using a two-way repeated measures ANOVA, with main factors of intensity (high, low), and video (INDEX, STATIC). In experiment 2 the normalised MEP amplitudes were submitted to a 3-way repeated measures ANOVA with main factors of muscle (FDI, ADM), OSP (FDI, ADM), and video (INDEX, LITTLE, STATIC). Significant interactions were further analysed through two separate ANOVAs for each muscle, with video as the main factor. For post-hoc comparisons, multiple pairwise t-tests with Sidak's correction were performed. The level of statistical significance for all analyses was set to α = 0.05. Effect sizes (ES) were reported as the difference in *z*-scores. This is equivalent to Cohen's *d*, which is the standardised difference between two means.

**Table 1:** MEP amplitudes obtained in experiment 1. Values are in µV (mean ± S.D.).

	Low Intensity		High Intensity	
	Index	Static	Index	Static
FDI-Muscle	439±197	345±199	1249±543	1212±426

**Table 2:** MEP amplitudes obtained in experiment 2. Values are in µV (mean ± S.D.).

	FDI-OSP			ADM-OSP		
	Index	Little	Static	Index	Little	Static
FDI-Muscle	581±304	508±345	497±331	641±572	527±554	564±563
ADM-Muscle	247±319	263±309	167±202	359±213	408±235	229±116

## Results

**Figure 2:**
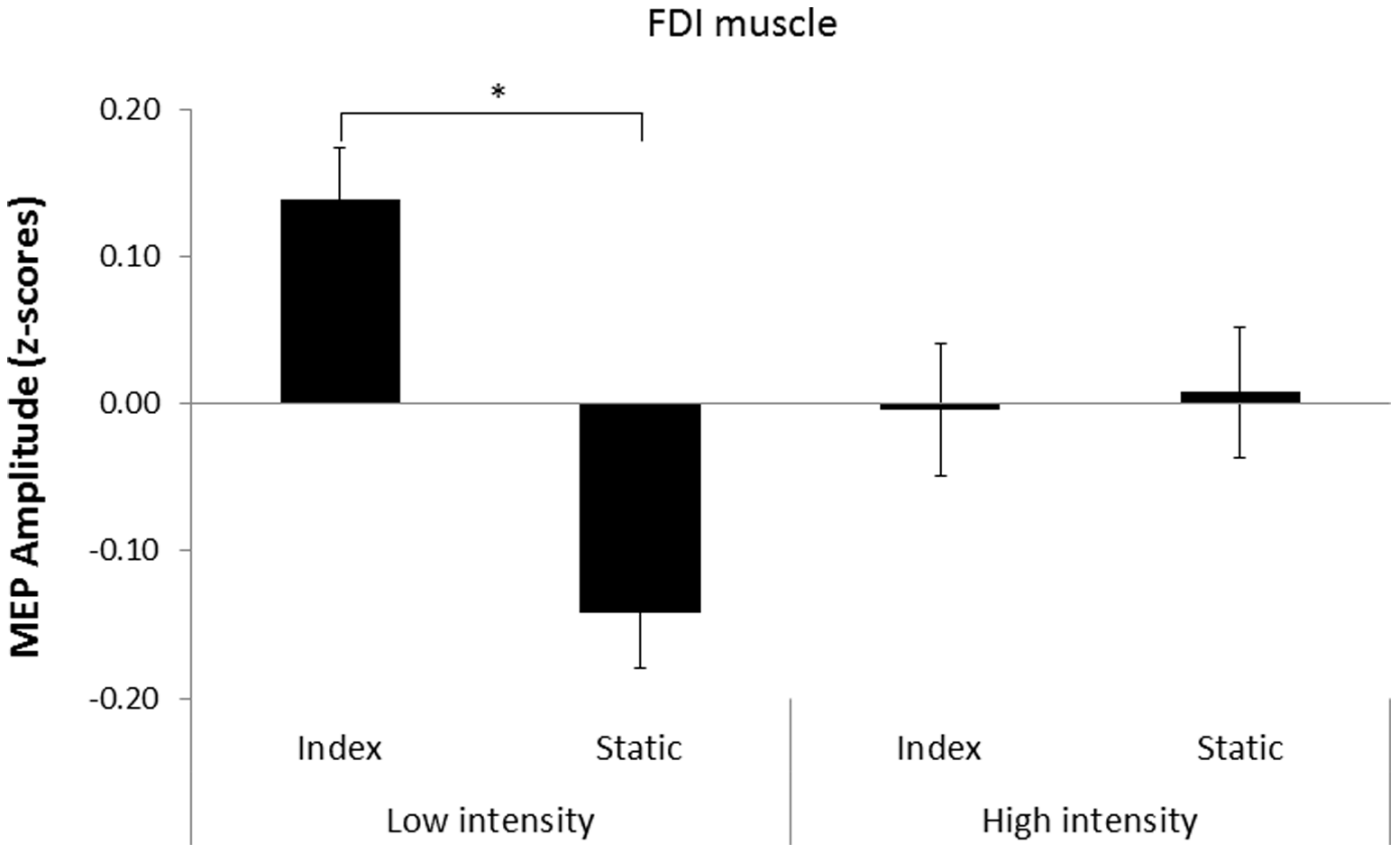
The mean MEP amplitudes recorded from the right FDI muscle during observation of index and static videos at high and low stimulation intensity in experiment 1. The MEP amplitudes are presented as *z*-scores (mean ± SE). Significant differences are indicated by asterisks (*p = 0.001).

The aim of experiment 1 was to test whether the magnitude of the motor facilitation recorded during action observation was affected by the TMS intensity. The repeated measures ANOVA revealed a significant video x intensity interaction, F(1,16) = 11.3, *p* = 0.004. Pairwise comparisons showed MEP amplitudes recorded from the FDI muscle were significantly higher during observation of INDEX as compared to STATIC (*p* = 0.001, ES = 0.28) with the use of low intensity TMS (see Figure 2). This effect was not present with the use of high intensity TMS (*p* = 0.89, ES = 0.01).

Experiment 2 tested whether action observation produced motor resonance effects, and if the choice of OSP influenced the magnitude of these effects. The most common FDI-OSP was 4 cm lateral and 1.5 cm anterior, relative to Cz (apex of the skull), compared to 4 cm lateral from Cz for the ADM-OSP. The mean RMT for the FDI-OSP was 47% (±9), with the ADM-OSP significantly higher at 50% (±9), *t* (18) = 2.5, *p* = 0.02 (Table 3).

**Table 3:** Individual participant's values for OSP and resting motor threshold percentage.

	FDI		ADM	
Participant	OSP	Threshold %	OSP	Threshold %
1	4 cm, 1.5 cm	45	4 cm, 1 cm	46
2	4 cm, 1 cm	39	4 cm, 1 cm	44
3	4 cm, −1 cm	44	4 cm, −1 cm	47
4	4 cm, 1.5 cm	67	5 cm, 0 cm	72
5	4 cm, 1 cm	42	5 cm, −1 cm	44
6	4 cm, 1.5 cm	43	4 cm, 1.5 cm	52
7	4 cm, 1.5 cm	56	4 cm, 0 cm	51
8	4 cm, 0 cm	57	5 cm, 2 cm	65
9	4 cm, 1.5 cm	37	4 cm, 0 cm	39
10	3 cm, 1.5 cm	55	4 cm, 0 cm	55
11	4 cm, 0.5 cm	38	4 cm, 0 cm	39
12	4 cm, −1 cm	55	3 cm, 0.5 cm	53
13	4 cm, 0.5 cm	58	5 cm, 1 cm	58
14	4 cm, 0 cm	38	4 cm, 1 cm	42
15	4 cm, 1.5 cm	39	4 cm, 0 cm	45
16	4 cm, 1.5 cm	47	5 cm, 0 cm	55
17	3 cm, 1.5 cm	42	3 cm, −1 cm	40
18	4 cm, 1 cm	50	3 cm, 0 cm	51
19	4 cm, 1 cm	48	4 cm, 0 cm	45

The most common OSPs were 4 cm lateral and 1.5 cm anterior for FDI-OSP, and 4 cm lateral for ADM-OSP (all relative to Cz). The mean threshold value was 47% for FDI-OSP and 50% for ADM-OSP.

**Figure 3:**
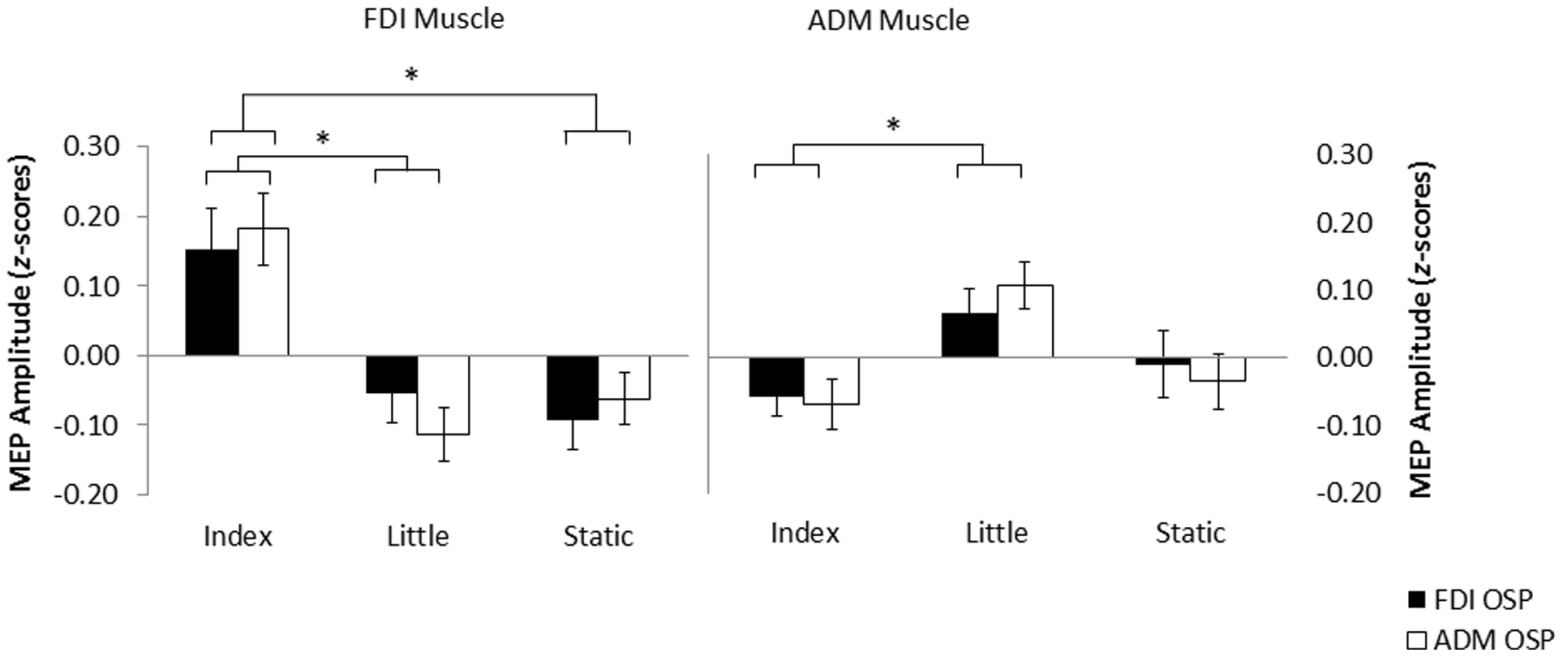
The mean MEP amplitudes recorded from the participants' right FDI (left panel) and right ADM (right panel) muscles during observation of index, little, and static videos recorded from the FDI-OSP (white) and ADM-OSP (black) in experiment 2. The MEP amplitudes are presented as *z*-scores (mean ± SE). Significant differences are indicated by asterisks (*p = 0.05).

The repeated measures ANOVA indicated that interactions of OSP x muscle, F(1,18) = 0.3, *p* = 0.57, OSP x video, F(2, 36) = 0.1, *p* = 0.93, and OSP x muscle x video, F(2,36) = 0.04, p = 0.24) were all non-significant. This indicated that the OSP location had no significant effect on the MEPs recorded during observation of the three video conditions for both the FDI and ADM muscles (see Figure 3). There was, however, a significant muscle x video interaction, F(2,36) = 14.7, *p*<0.001, demonstrating that a change in MEP amplitude across video conditions was dependent on the recorded muscle. Follow-up one way ANOVAs, with video as the main factor, were performed for both muscles. This showed a significant effect of video for both the FDI muscle, F(2,36) = 8.0, *p* = 0.001, and the ADM muscle, F(2,36) = 4.1, *p* = 0.03. Pairwise comparisons using Sidak's corrections showed MEP amplitudes recorded from the FDI muscle were significantly higher during the observation of INDEX as compared to both the LITTLE (*p* = 0.02, ES = 0.18) and STATIC (*p* = 0.02, ES = 0.23) videos. There was no significant difference in the MEP amplitudes recorded from the FDI during the observation of the STATIC and LITTLE videos (*p* = 0.99, ES = 0.05). MEP amplitudes recorded from the ADM muscle were significantly higher during observation of the LITTLE videos as compared to the INDEX videos (*p* = 0.01, ES = 0.21). MEP amplitudes recorded from the ADM muscle during observation of the STATIC videos were not significantly different from either the LITTLE (*p* = 0.12, ES = 0.14) or INDEX (*p* = 0.89, ES = 0.01) videos.

## Discussion

The results presented here show that observing another person's actions increases the excitability of the observer's motor system and that this effect is selective to those muscles that would be involved in the execution of the observed actions. To the best of our knowledge, this is the first study to investigate the influence of the choice of OSP and the intensity of TMS applied over M1. Although the choice of OSP did not significantly influence the magnitude of the MEP facilitation recorded during action observation, our current results clearly demonstrate they were only present when using low-intensity TMS.

MEP facilitations resulting from single-pulse TMS are considered evidence of motor resonance effects if they are specific to those muscles that are active during the execution of the observed action [10]. This requires that stable MEPs are evoked simultaneously in multiple muscles, which may be problematic since different muscles have their own OSP and motor threshold. The results of experiment 2 showed that MEP amplitudes recorded from the FDI muscle were facilitated during the observation of index-finger abductions as compared to the observation of little-finger abductions and the static control images. Similarly, MEP amplitudes recorded from the ADM muscle were facilitated during the observation of little-finger abductions as compared to index-finger abductions. These results indicate that action observation had a muscle specific effect on corticospinal excitability. Slightly inconsistent with this interpretation is that although the MEP amplitudes recorded from the ADM during the observation of little-finger abductions were higher than those recorded during the static control condition; this change was not statistically significantly, perhaps indicating that we did not have sufficient power to detect small effect sizes (∼0.14) with the number of participants tested.

Many studies have reported muscle-specific facilitation effects during action observation (e.g., [10], [19], [24], [27]), however, our current study was the first to investigate whether the choice of OSP modulated these effects. The common practice of determining the OSP for only the main muscle of interest assumes that the cortical representations of the tested muscles are stimulated in a similar manner from a single location. As shown in Table 3, some participants displayed a large difference in the location of the OSPs for the FDI and ADM muscles, such that the typical FDI-OSP was located 1.5 cm anterior to the most common ADM-OSP. Despite this difference in hotspot location, we did not detect any significant effect of OSP on the motor resonance effects recorded during action observation. This finding is encouraging for two reasons. First, it leads to the conclusion that previous studies reporting MEP facilitations specific to those muscles primarily involved in performing the observed action were unlikely to be confounded by the use of a single OSP despite eliciting MEPs in multiple muscles. Second, it allows researchers to test for motor facilitation effects in multiple muscles during a single experimental session. Participants can therefore undergo less experimental trials, which will reduce potential negative side-effects and lower dropout rates. It is important to note, however, that although the cortical representation of different finger muscles overlap within M1, those of arm and finger muscles may be considerably further apart [28], [29], therefore the validity of a single OSP for a comparison of these muscles requires additional investigation.

Motor resonance effects, as reported in the current experiments, are typically proposed to occur from the activity of MN regions enhancing M1 excitability via excitatory cortico-cortical connections [11]. Ventral premotor (PMv) and posterior parietal cortex (PPC), regions where MNs were originally discovered in the macaque monkey [5], and considered core parts of the human MNS [8], [9], are good candidates for mediating such effects. For example, PMv has strong reciprocal cortico-cortical connections with M1 that allow it to influence the amplitude of activity evoked by M1 stimulation [30]. Human evidence for an important role of MNS in producing motor resonance effects has been provided using a variety of different TMS techniques. For example, the application of 1 Hz repetitive TMS over the PMv, a ‘virtual lesion’ approach which can transiently inhibit the excitability of the underlying cortex [31], abolished motor resonance effects during the subsequent observation of index-finger abductions [13]. Further support for this hypothesis has been provided by twin-coil TMS experiments showing that both the PPC-M1 and the PMv-M1 pathways, important mediators of the control of grasping [32], [33], also show excitability modulations during the observation of hand actions [34].

The application of single-pulse TMS over M1, as performed in the current experiments, elicits a repetitive discharge of corticospinal volleys. The direct activation of corticospinal axons produces an early D-wave, which is then followed by a series of I-waves resulting from the indirect trans-synaptic activation of corticospinal neurons [15]. The type of stimulation can be controlled, to some extent, by the choice of stimulus intensity. With the coil orientation we used in the current study, low intensity TMS preferentially activates M1 in a trans-synaptic manner, whereas the direct activation of corticospinal axons occurs more readily at high stimulation intensities [15]. As summarised above, motor resonance effects are considered to reflect activity in MN regions modulating M1 activity through cortico-cortical pathways, the excitability of which will be reflected in I-wave amplitudes recruited by the TMS pulse. For this reason we hypothesised that motor resonance effects would be more prominent with low-intensity stimulation. D-waves result from the direct activation of corticospinal axons and therefore should be relatively unaffected by excitability changes induced by MN activity in cortico-cortical pathways. Our current results support this hypothesis as we detected a significant increase in corticospinal excitability during the observation of index-finger abductions with the use of low-intensity TMS (110% RMT) but not high intensity TMS (130% RMT). To a limited extent we can discount changes in spinal excitability since EMG activity was comparable between observation conditions. This finding is similar to Koch et al. [34] who found no change in corticospinal excitability during the observation of reach and grasp actions with high intensity TMS, but did not test with low intensity TMS.

Although our current results support the view that motor resonance effects elicited by action observation are mediated by indirect cortico-cortical connections from presumed MN regions, they appear incongruent with previous studies which have reported the presence of motor resonance effects following the application of high intensity TMS over M1 (e.g., [18], [19], [27], [35], [36]). Differences in experimental designs, for example, the lack of static conditions [35], [36], and the use of different observation conditions such as basketball actions [18], or wrist movements [35], [36] may lead to the contrasting results. The studies of Romani et al. [19] and Urgesi et al. [27] both utilised static controls and index-finger abductions as observation conditions and so we suggest that there are two other main factors which may explain the discrepancy in our results. First, instead of conducting the static and action conditions in separate blocks of trials [19], [27], we randomly interspersed these trials across all blocks, because ‘baseline’ measures of corticospinal excitability have been shown to fluctuate significantly depending on whether they were measured separately from or during observation blocks, perhaps due to change in attentional demands or movement of the stimulating coil [37]. Second, in addition to stimulation intensity, the coil orientation and current pulse waveform determine the manner in which TMS activates M1. The Magstim 200^2^ used in the current study provides monophasic stimulation, whereas the Magstim Rapid used by Romani et al. [19] and Urgesi et al. [27] provides biphasic stimulation. Monophasic stimulation is most effective when the induced current travels across M1 in a postero-anterior direction perpendicular to the central sulcus [22], [38]. This is opposite to the preferred direction for biphasic stimulation of the hand area [39]. When using a postero-anterior orientation, biphasic stimulation produces a more complex pattern of activation than monophasic stimulation [40]. For these reasons we suggest that the discrepancy between our results with high-intensity TMS may be due to the activation of different pathways resulting from the different pulse waveforms. This hypothesis could be tested by directly comparing the motor resonance effects elicited by the two stimulators during the observation of identical action observation conditions.

In summary, our current results indicate that small changes in the site of the OSP for two different finger muscles has a negligible effect on the presence of motor resonance effects elicited by the observation of simple hand actions. In contrast, a facilitation of MEP amplitude in the FDI muscle during the observation of index-finger abductions was detected using low-intensity but not high-intensity TMS. This latter finding fits with the view that MN activity elicited by the observation of other people's actions enhances M1 excitability via cortico-cortical pathways. The work presented in this paper provides a solid framework for which to explore the neural processes underlying action observation, which will help inform the design of observational learning paradigms as applied in clinical settings, such as stroke rehabilitation (e.g., [2]–[4]).

## References

[1] Rizzolatti G, Fogassi L, Gallese V (2001) Neurophysiological mechanisms underlying the understanding and imitation of action. Nat Rev Neurosci 2: 661–670. 11533734 10.1038/35090060

[2] Celnik P, Webster B, Glasser DM, Cohen LG (2008) Effects of action observation on physical training after stroke. Stroke 39: 1814–1820. 18403746 10.1161/STROKEAHA.107.508184 PMC3638075

[3] Ertelt D, Small S, Solodkin A, Dettmers C, McNamara A, et al (2007) Action observation has a positive impact on rehabilitation of motor deficits after stroke. Neuroimage 36 Suppl 2: T164–173. 17499164 10.1016/j.neuroimage.2007.03.043

[4] Holmes PS, Ewan L (2007) The use of structured observation as a stroke rehabilitation aid: an opinion from neuroscience. Br J Occup Ther 70: 454–456.

[5] Rizzolatti G, Fadiga L, Gallese V, Fogassi L (1996) Premotor cortex and the recognition of motor actions. Cog Brain Res 3: 131–141. 10.1016/0926-6410(95)00038-0 8713554

[6] Gallese V, Keysers C, Rizzolatti G (2004) A unifying view of the basis of social cognition. Trends Cogn Sci 8: 396–403. 15350240 10.1016/j.tics.2004.07.002

[7] Jeannerod M (1994) The representing brain. Neural correlates of motor intention and imagery. Behav Brain Sci 17: 187–245.

[8] Caspers S, Zilles K, Laird AR, Eickhoff SB (2010) ALE meta-analysis of action observation and imitation in the human brain. Neuroimage 50: 1148–1167. 20056149 10.1016/j.neuroimage.2009.12.112 PMC4981639

[9] Grezes J, Decety J (2001) Functional anatomy of execution, mental simulation, observation, and verb generation of actions: a meta-analysis. Hum Brain Mapp 12: 1–19. 11198101 10.1002/1097-0193(200101)12:1<1::AID-HBM10>3.0.CO;2-V PMC6872039

[10] Fadiga L, Fogassi L, Pavesi G, Rizzolatti G (1995) Motor facilitation during action observation: a magnetic stimulation study. J Neurophysiol 73: 2608–2611. 7666169 10.1152/jn.1995.73.6.2608

[11] Fadiga L, Craighero L, Olivier E (2005) Human motor cortex excitability during the perception of others' action. Curr Opin Neurobiol 15: 213–218. 15831405 10.1016/j.conb.2005.03.013

[12] Loporto M, McAllister C, Williams J, Hardwick R, Holmes P (2011) Investigating central mechanisms underlying the effects of action observation and imagery through transcranial magnetic stimulation. J Motor Beh 43: 361–373. 10.1080/00222895.2011.604655 21861627

[13] Avenanti A, Bolognini N, Maravita A, Aglioti SM (2007) Somatic and motor components of action simulation. Curr Biol 17: 2129–2135. 18083517 10.1016/j.cub.2007.11.045

[14] Day BL, Dressler D, Maertens de Noordhout A, Marsden CD, Nakashima K, et al (1989) Electric and magnetic stimulation of human motor cortex: surface EMG and single motor unit responses. J Physiol 412: 449–473. 2489409 10.1113/jphysiol.1989.sp017626 PMC1190586

[15] Di Lazzaro V, Oliviero A, Pilato F, Saturno E, Dileone M, et al (2004) The physiological basis of transcranial motor cortex stimulation in conscious humans. Clin Neurophysiol 115: 255–266. 14744565 10.1016/j.clinph.2003.10.009

[16] Gangitano M, Mottaghy FM, Pascual-Leone A (2004) Modulation of premotor mirror neuron activity during observation of unpredictable grasping movements. Eur J Neurosci 20: 2193–2202. 15450099 10.1111/j.1460-9568.2004.03655.x

[17] Montagna M, Cerri G, Borroni P, Baldissera F (2005) Excitability changes in human corticospinal projections to muscles moving hand and fingers while viewing a reaching and grasping action. Eur J Neurosci 22: 1513–1520. 16190904 10.1111/j.1460-9568.2005.04336.x

[18] Aglioti SM, Cesari P, Romani M, Urgesi C (2008) Action anticipation and motor resonance in elite basketball players. Nat Neurosci 11: 1109–1116. 19160510 10.1038/nn.2182

[19] Romani M, Cesari P, Urgesi C, Facchini S, Aglioti SM (2005) Motor facilitation of the human cortico-spinal system during observation of bio-mechanically impossible movements. Neuroimage 26: 755–763. 15955484 10.1016/j.neuroimage.2005.02.027

[20] Keel JC, Smith MJ, Wassermann EM (2001) A safety screening questionnaire for transcranial magnetic stimulation. Clin Neurophysiol 112: 720. 11332408 10.1016/s1388-2457(00)00518-6

[21] Oldfield RC (1971) The assessment and analysis of handedness: the Edinburgh inventory. Neuropsychologia 9: 97–113. 5146491 10.1016/0028-3932(71)90067-4

[22] Brasil-Neto JP, Cohen LG, Panizza M, Nilsson J, Roth BJ, et al (1992) Optimal focal transcranial magnetic activation of the human motor cortex: effects of coil orientation, shape of the induced current pulse, and stimulus intensity. J Clin Neurophysiol 9: 132–136. 1552001

[23] Clark S, Tremblay F, Ste-Marie D (2003) Differential modulation of corticospinal excitability during observation, mental imagery and imitation of hand actions. Neuropsychologia 42: 105–112. 10.1016/s0028-3932(03)00144-1 14615080

[24] Loporto M, McAllister C, Edwards MG, Wright DJ, Holmes PS (2012) Prior action execution has no effect on corticospinal facilitation during action observation. J Motor Behav 43: 361–373. 10.1016/j.bbr.2012.03.009 22449863

[25] Williams J, Pearce AJ, Loporto M, Morris T, Holmes PS (2012) The relationship between corticospinal excitability during motor imagery and motor imagery ability. Beh Brain Res 226: 369–375. 10.1016/j.bbr.2011.09.014 21939692

[26] Rossini PM, Barker AT, Berardelli A, Caramia MD, Caruso G, et al (1994) Non-invasive electrical and magnetic stimulation of the brain, spinal cord and roots: basic principles and procedures for routine clinical application. Report of an IFCN committee. Electroencephalogr Clin Neurophysiol 91: 79–92. 7519144 10.1016/0013-4694(94)90029-9

[27] Urgesi C, Candidi M, Fabbro F, Romani M, Aglioti SM (2006) Motor facilitation during action observation: topographic mapping of the target muscle and influence of the onlooker's posture. Eur J Neurosci 23: 2522–2530. 16706859 10.1111/j.1460-9568.2006.04772.x

[28] Melgari J, Pasqualetti P, Pauri F, Rossini PM (2008) Muscles in “concert”: Study of primary motor cortex upper limb functional topography. PLoS One 3: e3069. 18728785 10.1371/journal.pone.0003069 PMC2518106

[29] Sanes JN, Donoghue JP (2000) Plasticity and primary motor cortex. Annu Rev Neurosci 23: 393–415. 10845069 10.1146/annurev.neuro.23.1.393

[30] Shimazu H, Maier M, Cerri G, Kirkwood P, Lemon RN (2004) Macaque ventral premotor cortex exerts powerful facilitation of motor cortex outputs to upper limb motoneurons. J Neurosci 24: 1200–1211. 14762138 10.1523/JNEUROSCI.4731-03.2004 PMC6793573

[31] Chen R, Classen J, Gerloff C, Celnik P, Wassermann EM, et al (1997) Depression of motor cortex excitability by low-frequency transcranial magnetic stimulation. Neurology 48: 1398–1403. 9153480 10.1212/wnl.48.5.1398

[32] Davare M, Lemon R, Olivier E (2008) Selective modulation of interactions between ventral premotor cortex and primary motor cortex during precision grasping in humans. J Physiol 586: 2735–2742. 18403420 10.1113/jphysiol.2008.152603 PMC2536583

[33] Koch G, Fernandez Del Olmo M, Cheeran B, Ruge D, Schippling S, et al (2007) Focal stimulation of the posterior parietal cortex increases the excitability of the ipsilateral motor cortex. J Neurosci 27: 6815–6822. 17581969 10.1523/JNEUROSCI.0598-07.2007 PMC6672690

[34] Koch G, Versace V, Bonni S, Lupo F, Lo Gerfo E, et al (2010) Resonance of cortico-cortical connections of the motor system with the observation of goal directed grasping movements. Neuropsychologia 48: 3513–3520. 20691198 10.1016/j.neuropsychologia.2010.07.037

[35] Alaerts K, Heremans E, Swinnen SP, Wenderoth N (2009) How are observed actions mapped to the observer's motor system? Influence of posture and perspective. Neuropsychologia 47: 415–422. 18926836 10.1016/j.neuropsychologia.2008.09.012

[36] Alaerts K, Swinnen SP, Wenderoth N (2009) Is the human primary motor cortex activated by muscular or direction-dependent features of observed movements? Cortex 45: 1148–1155. 19100971 10.1016/j.cortex.2008.10.005

[37] Labruna L, Fernandez-del-Olmo M, Ivry RB (2011) Comparison of different baseline conditions in evaluating factors that influence motor cortex excitability. Brain Stim 4: 152–155. 10.1016/j.brs.2010.09.010 21777875

[38] Mills KR, Boniface SJ, Schubert M (1992) Magnetic brain stimulation with a double coil: the importance of coil orientation. Electroencephalogr Clin Neurophysiol 85: 17–21. 1371739 10.1016/0168-5597(92)90096-t

[39] Kammer T, Beck S, Thielscher A, Laubis-Herrmann U, Topka H (2001) Motor thresholds in humans: a transcranial magnetic stimulation study comparing different pulse waveforms, current directions and stimulator types. Clin Neurophysiol 112: 250–258. 11165526 10.1016/s1388-2457(00)00513-7

[40] Di Lazzaro V, Oliviero A, Mazzone P, Insola A, Pilato F, et al (2001) Comparison of descending volleys evoked by monophasic and biphasic magnetic stimulation of the motor cortex in conscious humans. Exp Brain Res 141: 121–127. 11685416 10.1007/s002210100863

